# Pharmacokinetic Characterisation and Comparison of Bioavailability of Intranasal Fentanyl, Transmucosal, and Intravenous Administration through a Three-Way Crossover Study in 24 Healthy Volunteers

**DOI:** 10.1155/2021/2887773

**Published:** 2021-11-29

**Authors:** S. Nardi-Hiebl, J. W. Ndieyira, Y. Al Enzi, W. Al Akkad, T. Koch, G. Geldner, C. Reyher, L. H. J. Eberhart

**Affiliations:** ^1^Department of Anaesthesia and Intensive Care, University Hospital Marburg, Marburg, Germany; ^2^Division of Medicine, University College London, London, UK; ^3^Department of Mathematics and Natural Science, Gulf University of Science and Technology, Mubarak Al-Abdullah, Kuwait; ^4^Royal Free London Hospital, University College London, London, UK; ^5^Department of Anaesthesiology Intensive Care Medicine Emergency Medicine and Pain Therapy, RKH Kliniken-Hospital Ludwigsburg, Ludwigsburg, Germany; ^6^Department of Anaesthesiology Intensive Care Medicine Emergency Medicine and Pain Therapy, Klinikum Kassel GmbH, Kassel, Germany

## Abstract

**Background:**

For more than 60 years, the synthetic opioid fentanyl has been widely used in anaesthesia and analgesia. While the intravenous formulation is primarily used for general anaesthesia and intensive care settings, the drug's high lipophilic properties also allow various noninvasive routes of administration. Published data suggest that intranasal administration is also attractive for use as intranasal patient-controlled analgesia (PCA). A newly developed intranasal fentanyl formulation containing 47 *μ*g fentanyl, intravenous fentanyl, and oral transmucosal fentanyl citrate were characterised, and bioavailability was compared to assess the suitability of the intranasal formulation for an intranasal PCA product.

**Methods:**

27 healthy volunteers were enrolled in a single-centre, open-label, randomised (order of treatments), single-dose study in a three-period crossover design. The pharmacokinetics of one intranasal puff of fentanyl formulation (47 *μ*g, 140 mL per puff), one short intravenous infusion of 50 *μ*g fentanyl, and one lozenge with an integrated applicator (200 *μ*g fentanyl) were studied, and bioavailability was calculated. Blood samples were collected over 12 hours, and plasma concentrations of fentanyl were determined by HPLC with MS/MS detection.

**Results:**

24 volunteers completed the study. The geometric mean of AUC_0-tlast_ was the highest with oral transmucosal administration (1106 h *∗* pg/ml, CV% = 32.86), followed by intravenous (672 h *∗* pg/ml, CV% = 32.18) and intranasal administration (515 h *∗* pg/ml, CV% = 30.10). *C*_max_ was 886 pg/ml (CV% = 59.38) for intravenous, 338 pg/ml (CV% = 45.61) for intranasal, and 310 pg/ml (CV% = 29.58) for oral transmucosal administration. *t*_max_ was shortest for intravenous administration (0.06 h, SD = 0.056), followed by intranasal (0.21 h, SD = 0.078) and oral transmucosal administration (1.20 h, SD = 0.763). Dose-adjusted absolute bioavailability was determined to be 74.70% for the intranasal formulation and 41.25% for the oral transmucosal product. In total, 38 adverse events (AEs) occurred. Fourteen AEs were potentially related to the investigational items. No serious AE occurred.

**Conclusion:**

Pharmacokinetic parameters and bioavailability of the investigated intranasal fentanyl indicated suitability for its intended use as an intranasal PCA option.

## 1. Introduction

Fentanyl has been used in anaesthesiology and analgesia for almost 60 years. First synthesised by Paul Janssen in the 1960s, it was introduced in anaesthesia and intensive care medicine as the first high potent µ-receptor agonist with an analgesic potency approximately 100 times higher than morphine [1]. Due to its high lipophilic properties, fentanyl is rapidly distributed and thus offers a relative short-lived analgesic effect of around 30 to 60 minutes. Through multiple or continuous administrations, it can accumulate over time [2]. Either fentanyl base or citrate salt is used for pharmaceutical formulations, whereby the base is virtually water-insoluble. The generally accepted explanation for the metabolisation of fentanyl is through N-dealkylation to norfentanyl by the cytochrome P450 (CYP) 3A4 [3–5], whereby none of the metabolites are considered to be pharmacologically active in a relevant manner [6]. It has been suggested that other unknown metabolic routes play an important role [7]. As a potent opioid, it possesses all the opioid-inherent adverse effects: nausea, vomiting, urinary retention, and pruritus, as well as potential respiratory depression as the most severe consequence [8].

Pharmacological developments over the last 20 years have led to new formulations and drug delivery technologies allowing the noninvasive administration of fentanyl. Such products offer individually adjusted titration and duration of action for a defined episode with a quick onset intended to treat chronic and acute pain states [9–12]. The intravenous formulation is exclusively used in anaesthesia and intensive care as high peak concentrations rapidly lead to acute respiratory depression. On the opposite end of the spectrum, transdermal delivery systems provide stable plasma concentrations with a slow onset qualifying such devices to treat chronic pain. Oral transmucosal and intranasal formulations are commonly utilised for breakthrough pain management for patients on chronic opioid intake. No intranasal delivery systems have been available for fentanyl to be used for opioid-naive patients. Due to the potential issues concerning misuse and abuse of fentanyl, dose control and patient authentication are mandatory safety features.

The intranasal administration route provides various benefits such as ease of use and noninvasiveness. As the nasal cavity comprises a large surface and the turbinate structures support the exposure of the mucosal surface and the inhaled air, absorption of drug compounds is rapid, and the well-perfused epithelial membrane further supports this effect. In addition, small lipophilic compounds such as fentanyl are usually well absorbed from mucosal surfaces, and as such, the onset is often clinically comparable with intravenous injection [13]. Intranasal administration has an added benefit in improving bioavailability by avoiding the enterohepatic first-pass effect since venous outflow of nasal mucosa directly enters systemic circulation [14]. Previous studies with intranasal fentanyl have reported high values for bioavailability, a shorter period to reach maximum concentrations, and faster pain relief compared with other routes of noninvasive administrations [15–18].

Intranasal administration of fentanyl has been systematically studied for use in postoperative pain management, also in the context of intravenous patient-controlled analgesia (i.v. PCA). Patients receiving i.v. PCA report higher satisfaction and better pain control than patients receiving non-patient-controlled analgesia [19]; however, utilisation might not match the necessity to provide the best patient care [20]. One reason for underutilisation is the complexity around i.v. PCA [21, 22].

Over the last years, the community has seen two interesting technologies for postoperative pain management: a disposable electronic transdermal system (IONSYS®, The Medicines Company, Parsippany/New Jersey, USA) [10] and a dispenser for sublingual tablets in combination with sufentanil-containing bioadhesive nanotablets (Zalviso®, Grünenthal GmbH, Aachen, Germany) [23]. These devices offered noninvasive drug delivery without the need for a catheter, consequently a streamlined process and advantages in postoperative mobilisation. However, both products were not commercially viable.

Current development focuses on intranasal PCA as an effective alternative to i.v. PCA while offering the benefits of noninvasive administration [24, 25] with similar satisfaction to i.v. PCA [26]. Although first reported more than 30 years ago, there is no commercial PCA product available incorporating the advantages of intranasal fentanyl for postoperative pain management. The recently developed technical concept for intranasal administration incorporates features for safe postoperative pain management and thus might overcome the challenges previously faced [27].

As part of this development, the characterisation of fentanyl absorption and bioavailability for an intranasal fentanyl formulation and two other fentanyl preparations with different routes of administration are hereby presented. The objective of this study was to investigate and compare the bioavailability of the new formulation containing 47 *μ*g fentanyl (74 *μ*g fentanyl citrate) and 0.1% sodium hyaluronate (added for better tolerability) in 140 *μ*l per spray puff with intravenous and oral transmucosal administration through a three-way crossover study in 24 healthy subjects.

## 2. Materials and Methods

After the Institutional Ethics Committee approval, the study was registered with the European Union Drug Regulating Authorities Clinical Trials Database (No. 003034-17) and subsequently conducted per protocol, the principles of the Declaration of Helsinki, ICH-GCP guidelines, and the requirements of the German Drug Law [28, 29].

### 2.1. Volunteer Selection

Twenty-seven healthy nonsmoking men and women aged between 18 and 55 years with a body mass index between 18 and 27 kg/m^2^ were recruited, thereof 15 women and 12 men. All volunteers provided written informed consent. All study participants were in good health. Screening at commencement and with the conclusion of the study was conducted according to protocol. Screening included the medical history, physical examination, clinical laboratory tests, 12-lead ECG, pregnancy testing for women, and alcohol and drug screening. No clinically relevant deviations from standard results or laboratory findings were found. The physical examination, clinical laboratory tests, and ECG were repeated at the end of the study. At the beginning of each study period, vital parameters were assessed, and before administration, one and 12 hours after administration, safety laboratory parameters (SGPT/ALAT, SGOT/ASAT, *γ*-GT) were determined. Tolerability monitoring included periodic measurement of vital signs and recording of adverse events.

### 2.2. Study Design

This study was performed at a phase I unit as an open-label, randomised (order of treatments), single-dose study in a three-period crossover design within a timeframe of four weeks with at least a three-day wash-out period between each period.

The investigational items were administered either as a single intranasal puff of the new fentanyl formulation (47 *μ*g fentanyl, 140 *μ*L per puff) (INTRANASAL), a short intravenous infusion of 50 *μ*g fentanyl (1 mL) (INTRAVENOUS), or one lozenge with an integrated applicator (oral transmucosal fentanyl citrate—200 *μ*g fentanyl) (TRANSMUCOSAL).

To reduce the risk of pronounced respiratory depression or effects of unreported drug dependence before this study, naltrexone was coadministered at all three time points. The volunteers received orally 25 mg naltrexone hydrochloride the evening before study day, followed by observation of one hour for withdrawal symptoms. Further, 50 mg naltrexone hydrochloride per os was administered three hours before administration of fentanyl. While volunteers resided in the study confinement, intake of food and beverages was standardised. All volunteers fastened at least 10 hours before administration of any investigational item. Only drinking water was allowed up to four hours before administration except for 150 mL water for the intake of naloxone three hours before administration. On the study day, the volunteers received standardised meals at three, six, and 12 hours after administration.

### 2.3. Sampling and Data Collection

Before the commencement of the first administration, each volunteer was randomly assigned to one of the six sequences (full permutation).

Before receiving intranasal administration, the respective volunteer was asked to clean up the nose by carefully blowing to avoid malabsorption due to mucous residues. Any sign of rhinitis, nose bleeding, blocked nose, or other specifics that could impair the absorption could lead to exclusion for intranasal administration. After administering the spray puff, the volunteer was instructed to avoid any manipulation on the nose for one hour. Volunteers prepared for intravenous administration received the study medication diluted in 10 mL of 0.9% sodium chloride as a short infusion facilitated by a syringe pump for 2 minutes. In the case of the lozenge, the oral cavity of the volunteer was assessed for any signs of mucous lesions, inflammations, specific or global oral pain incidents, and other specifics, which may result in the exclusion. Within five to 15 minutes before administering, the volunteer was requested to clean and moisten the oral cavity by taking a sip of water. For administration purposes, the volunteer moved the lozenge rotating along the cheeks for up to 15 minutes until the entire drug-containing matrix was dissolved.

According to the investigational item, blood sampling took place using an intravenous cannula and was performed in an adopted schedule. For nasal administration, samples were taken before and 3 min, 6 min, 9 min, 12 min, 15 min, 20 min, 25 min, 30 min, 45 min, 60 min, 80 min, 100 min, 120 min, 2.5 h, 3 h, 4 h, 5 h, 6 h, 8 h, and 12 h (21 samples) after administration, for intravenous administration, samples were taken prior to and 2 min (end of infusion), 5 min, 8 min, 11 min, 15 min, 20 min, 30 min, 45 min, 60 min, 80 min, 100 min, 120 min, 2.5 h, 3 h, 4 h, 5 h, 6 h, 8 h, and 12 h (20 samples) after administration, and for oral transmucosal administration, samples were taken prior to and 5 min, 10 min, 15 min (the latest at the end of sucking), 20 min, 25 min, 30 min, 45 min, 60 min, 80 min, 100 min, 120 min, 2.5 h, 3 h, 3.5 h, 4 h, 5 h, 6 h, 8 h, and 12 h (20 samples) after administration.

The blood samples were taken using heparinised 7.5 mL tubes (Monovette, Sarstedt) and were centrifuged within 30 minutes of withdrawal (2000 g, 10 minutes, 4°C). The supernatant plasma was frozen latest 60 minutes after sampling at −20°C and stored in a frozen state until called for analysis.

### 2.4. Analytical Procedures

Fentanyl analysis was based on a solid-phase extraction from plasma followed by its determination using high-performance liquid chromatography (HPLC) in which the HPLC method was coupled to MS/MS detector (Agilent Technologies, MicroMass) with analytical columns (Polaris C18-A, 5 *μ*m, 100 × 3.0 mm).

To extract fentanyl, 500 *µ*L plasma of each sample was pipetted per well into a 96-well plate, and 500 *µ*L 2.5% acetic acid (containing the IS) was added. The well plate was shaken for 5 minutes at 1000 rpm. The samples were transferred onto a preconditioned (with 400 *µ*L methanol without vacuum and 400 *µ*L 0.1% acetic acid) MP1 SPEC plate. The samples were passed through the plate by low vacuum. The plate was washed with 400 *µ*L 0.1% acetic acid followed by 200 *µ*L methanol. Subsequently, the plate was dried by high vacuum for 10 minutes. The plate was then eluted four times with 100 *µ*L each of methanol/ammonia solution, and 10 *µ*L was injected. To determine the final fentanyl concentration, the experimental conditions consisted of 2 mM ammonium acetate buffer at pH 4.5 (Solvent A) and 0.1% formic acid in methanol (Solvent B) with a defined ratio of Solvent B/Solvent A fixed at 90% to 10% and mixed by HPLC pump during run with a flow rate of 0.2 mL/min and 5 min run time.

The peak area of transitions of m/z 337.2 ⇒ 188.3 was monitored during multiple reaction monitoring against m/z 342.2⇒188.3 for the internal standard fentanyl-d5. Three transitions were attached in the MS method to reduce crosstalk from the internal standard and reverse. The calculation of analyte concentration was obtained from the response of peak area of the analyte to the peak area of the internal standard, using a quadratic fit with 1/concentration weighting.

Before validating the plasma fentanyl concentration, first, the fentanyl standard solutions to be used for the calibration curves were prepared in blank plasma. 10 pg/mL fentanyl addition was set as the lower limit of quantitation, and the calibration curves were linear over the entire measurement range of 10 to 1000 pg/mL so that the sample measurements were effectively controlled by the calibration curves. For each concentration, the accuracy was characterised between ±13.1% (LLQC) and ±8.2% (HQC) with precision between ±8.1% (HQC) and ±11.5% (MQC), respectively, thus confirming that the resulting values were within acceptance criteria. Intra- and inter-day variations of quality control samples covering the intended concentration range demonstrated that accuracy and precision were well within the established acceptance criteria. For accuracy, the mean value was within 15% of the actual value, and for precision, the value at each concentration level did not exceed 15% of the coefficient of variation (CV). The standard approach of the US-FDA Guidance for Industry on Bioanalytical Method Validation was followed.

### 2.5. Pharmacokinetic Parameters

Pharmacokinetic parameters were determined model-independently for each treatment phase, and characteristics were derived directly from measured concentrations; noncompartmental analysis (NCA) was utilised. Actual sampling times were considered for evaluation.

The area under the concentration versus time curve from administration time to the last measurement time point with a concentration value above the lower limit of quantification (AUC_0-tlast_) was calculated using the linear trapezoidal rule up to *C*_max_ and subsequently the log trapezoidal rule for the remainder of the curve. AUC_0-∞_ was calculated as the sum of AUC_0-tlast_ and AUC_expol_, whereby AUC_expol_ was determined by the concentration at the last time quantifiable point (C_last_) divided by the apparent terminal elimination rate constant *λ*, calculated by log-linear regression analysis taking into account the baseline-corrected values above zero. AUC_expol%_ was calculated by multiplying AUC_expol_ by 100 and divided by AUC_0-∞_. Terminal half-life (*t*_1/2_) was calculated by dividing the natural logarithm of 2 by *λ*.

### 2.6. Statistical Evaluation

Descriptive statistics were presented for all determined pharmacokinetic parameters and adverse events without dose adjustment to present individual characterisation. We utilised the software applications Phoenix WinNonlin (Certara Companies), MedCalc (MedCalc Software), and Microsoft Excel (Microsoft Corporation) for calculations and analysis. Statistical analysis was performed with a significance level of 0.05. A blinded review of the results and general applicability of the statistical procedure on the study data set, identification of possible outliers, and decision upon exclusion of subjects from analysis or specific calculations were performed.

We conducted a pairwise statistical comparison of *C*_max_, *t*_max,_ and AUC_0-tlast_, whereby we used the two-sided *t*-test for *C*_max_ and AUC_0-tlast_ and the Mann–Whitney test for *t*_max_. The values for *C*_max_ and AUC AUC_0-tlast_ were found to be normally distributed (D'Agostino–Pearson test). For statistical comparison of bioavailability, we applied dose adjustment to the investigational items. Relative bioavailability was derived by the ratios of geometric means (point estimates). As interval estimates, 90% confidence intervals (CI) were determined by parametric analysis (two one-sided *t*-tests). While a multiplicative model was applied for all AUC and C_max_ analyses, *t*_max_ and *t*_1/2_ were analysed by employing an additive model. The factors considered for variance analysis for AUC_0-tlast_, AUC_0-∞,_ and *C*_max_ values were formulation, period, sequence, and volunteer (sequence). Intra-subject variabilities were estimated, and period, subject, and sequence effects were determined.

We concluded that obtaining data from 27 volunteering individuals was appropriate to meet the study's objective and the financial means allocated. Therefore, we did not calculate the sample size.

### 2.7. Adverse Events

Any pretreatment signs and symptoms (PTSS) and adverse events (AEs) reported were classified regarding severity and potential relationship with the administration of the investigational items. In the case of an AE, the volunteer was monitored and followed up until satisfactory recovery.

## 3. Results and Discussion

### 3.1. Results

#### 3.1.1. Study Population

24 of 27 enrolled volunteers completed the study (Table 1). One volunteer dropped out due to vomiting after intake of naltrexone, and another volunteer dropped out due to continuous vomiting after intake of the first investigational item. One further volunteer stated personal reasons for leaving the study after completing the first period.

While data from all 27 volunteers were included in the safety analysis, only the data of the 24 volunteers completing the study were used for pharmacokinetic analysis.

The study was conducted without significant protocol deviation. Even where minor deviations occurred, neither of them were judged as clinically or pharmacokinetically influential and relevant.

#### 3.1.2. Descriptive Statistics

**Figure 1:**
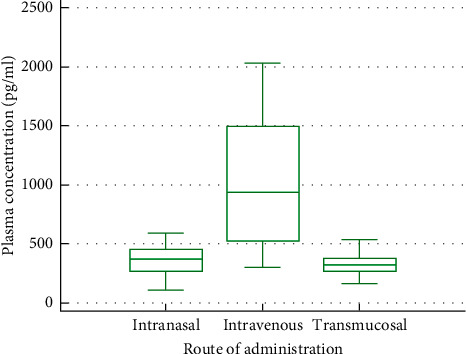
Box and whisker plot of *C*_max_ values.

Compared to INTRANASAL, INTRAVENOUS presented a significantly higher geometric mean of Cmax (Tables 2–4, Figure 1). INTRAVENOUS also presented a significantly higher *C*_max_ than TRANSMUCOSAL (Table 5), but no significant difference in *C*_max_ between INTRANASAL and TRANSMUCOSAL could be determined. In absolute values, INTRAVENOUS exhibited the highest level of *C*_max_ with 886 pg/ml (geometric mean, SD = 59.38).

Systemic exposure (AUC_0-tlast_) between INTRANASAL and INTRAVENOUS was significantly distinct, and the comparison of the AUC_0-tlast_ value of TRANSMUCOSAL with INTRANSAL and INTRAVENOUS indicated a significant difference, too.

**Figure 2:**
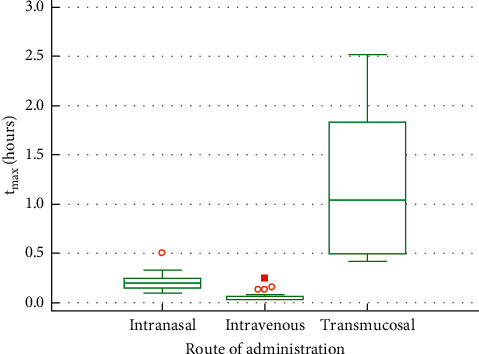
Box and whisker plot of *t*_max_ values.

The time to reach maximum drug concentration in plasma (*t*_max_) was significantly different between the compared items. INTRAVENOUS and INTRANASAL lead to a comparatively short *t*_max_ (0.06 h and 0.21 h); TRANSMUCOSAL exhibited a considerably higher t_max_ value (1.2 h, SD = 0.763) (Figure 2).

**Figure 3:**
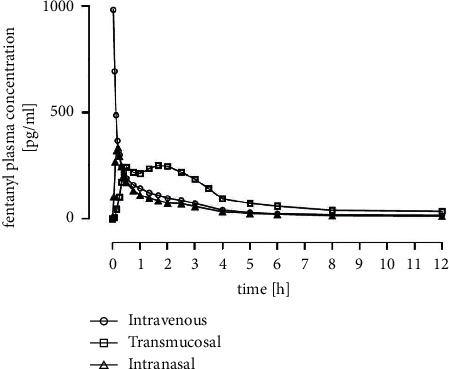
Overlay of mean plasma concentration versus time curves of fentanyl of INTRAVENOUS, TRANSMUCOSAL, and INTRANASAL.

The mean curve of INTRANASAL showed a steep and fast increase within the first minutes after administration peaking after 12 minutes with a constant decrease over time (Figure 3). TRANSMUCOSAL depicted a biphasic plasma concentration profile. INTRAVENOUS illustrated peak level at the first time point followed by a continuous decrease.

#### 3.1.3. Bioavailability Analysis

The values of AUC_0-tlast_ point estimates for INTRANASAL in comparison with the two other investigational items ranged between 45.27% and 198.18%. Absolute bioavailability (F_abs_) was determined at 74.70% (90% CI: 69.01–80.87, CV_ANOVA_: 16.10%, power: 99.74%) (Table 6).

Comparing INTRANASAL to non-dose-adjusted TRANSMUCOSAL leads to a relative bioavailability (F_rel_) of 45.27% (90% CI: 41.64–49.23, CV_ANOVA_: 17.00%, power: 99.54%), while adjusting the dose of TRANSMUCOSAL to 47 *μ*g resulted in F_rel_ of 198.18% (90% CI: 182.26–215.49, CV_ANOVA_: 17.40%, power: 99.54%).

*C*_max_ point estimates for dose-adjusted INTRANASAL compared to INTRAVENOUS were 37.22% (90% CI: 30.15–45.94, CV_ANOVA_: 44.50%, power: 51.13%), while that for dose-adjusted TRANSMUCOSAL compared to INTRAVENOUS was only 8.78% (90% CI: 7.02–10.99, CV_ANOVA_: 45.60%, power: 49.72%); however, both value calculations exhibited low power (Table 7).

#### 3.1.4. Adverse Events

In total, 17 of the 27 volunteers reported 38 adverse events (AEs) (Table 8). 31 AEs were classified as mild, while 7 AEs were classified as moderate. 14 of the 38 AEs appeared to have a possible relationship with the investigational products. Five AEs occurred after administering naltrexone hydrochloride. For 24 AEs, no causal relationship or classification could be established. None of the AEs were considered severe. All volunteers experiencing AEs recovered without any sequelae.

The five most common reported AEs were nausea (7 AEs), headache (5 AEs), and vomiting (3 AEs). Further AEs reported more than once included tiredness and stomach pain (2 AEs each).

In comparison with INTRAVENOUS and TRANSMUCOSAL, the administration of INTRANASAL resulted in the highest number of drug-related AEs (7 AEs versus 3 AEs versus 4 AEs). INTRANASAL also exhibited the highest total number of AEs (14 AEs versus 12 AEs versus 6 AEs).

From the list of the five most common reported AEs, headache (2 AEs), vomiting (2 AEs), and tiredness (2 AEs) were the most frequent events for INTRANASAL, nausea (4 AEs) for INTRAVENOUS, and headache (2 AEs) and nausea (2 AEs) for TRANSMUCOSAL.

### 3.2. Discussion

The study enrolled 27 healthy volunteers of both sexes in a monocentric, open-label, sequence randomised approach performed in a three-period changeover design whereby 24 volunteers completed. According to the randomisation plan, one of three investigational products were administered during each period either as a single intranasal spray puff of 140 *μ*L containing 47 *μ*g fentanyl, one intravenous infusion of 10 mL sodium chloride containing 50 *μ*g fentanyl over two minutes, or one lozenge with an integrated applicator for oral transmucosal absorption containing 200 *μ*g fentanyl administered over 15 minutes. To our knowledge, the data presented hereby represent the first publication of direct pharmacokinetic comparison for the three different routes of administration of fentanyl within the same study subjects.

The investigational item INTRAVENOUS was primarily chosen not only for pharmacokinetic but also for safety comparison reasons. The investigational product TRANSMUCOSAL had been selected because it is prescribed for pain episodes of acute pain in cancer patients and thus principally thought to be close to the potential indication of postoperative pain as the target indication for the INTRANASAL product. Therefore, it is anticipated that the comparison of maximum systemic exposure between INTRANASAL and TRANSMUCOSAL would indicate a potential comparable therapeutic effect for acute postoperative pain.

In previous studies, values for bioavailability and *C*_max_ for oral transmucosal fentanyl citrate of 50% and 0.4 ng/mL (arithmetic mean) were reported [30]. This study observed 41% and 310 pg/mL (geometric mean). Values between 55% and 71% for bioavailability and 0.36 ng/mL (median) for *C*_max_ were reported for a nasal spray containing 50 *μ*g fentanyl per puff [13], while values of 74.71% and 370 pg/mL were determined for INTRANASAL. However, one study reported a substantial lower *C*_max_ (180 pg/mL) while administering a slightly higher amount of intranasal fentanyl (54 *μ*g fentanyl per puff) [16]. The most probable explanation for this deviation is the analytical method used in this study, which employed a radioimmunoassay with a 40 pg/mL LOD to quantify fentanyl concentrations in plasma.

Differences in comparison with other studies [31–33] result from different sampling and observation times, which might not necessarily allow an accurate determination of *t*_1/2_ and subsequently lead to an underestimation of *t*_1/2_ and AUC while overestimating clearance (Cl). As fentanyl distributes rapidly, accurate and close sampling during the first minutes after administration is crucial. This study, therefore, sampled initially in a 3-minute interval for intravenous and intranasal administration up to 15 minutes.

Within the INTRAVENOUS and TRANSMUCOSAL groups, *C*_max_ and *t*_max_ values, respectively, exhibited comparatively high variations. This is consistent with previous findings that such pharmacokinetic parameters are occasionally highly variable between subjects for certain fentanyl formulations [34, 35]. In the case of the administration of intravenous fentanyl, a swift distribution into vascularised compartments and subsequently redistribution to fat and muscle tissue occur, while intranasal or oral administrated fentanyl is first distributed to local membranes before entering the systemic circulation [36]. Oral transmucosal absorption involves various factors such as the amount of salvia and has been described as complex [30]. The pharmacokinetic profile highly depends on the fraction absorbed by the oral mucosa, where around 25% of the available fentanyl is quickly absorbed and enters directly into circulation. The remaining fraction swallowed enters circulation later, but thereof around 50% is purged by enterohepatic and intestinal first-pass elimination [37]. This can also be noticed by the depicted biphasic pharmacokinetic profile for TRANSMUCOSAL. Individual constitution of the entire body and individual metabolism have therefore a considerable influence on the parameters. It cannot be assured that even in the same volunteer the characteristics of absorption and distribution of fentanyl remain comparable on the various different study days [30]. The variability of such values of intranasal fentanyl appears to be lower than other routes of administration; however, the importance of such variations of pharmacokinetic properties for clinical practice has apparently only a minor effect on efficacy or tolerability [38].

INTRANASAL exhibits only half the extent of exposure (AUC_0-tlast_) compared with TRANSMUCOSAL, which contains four times more fentanyl. Relative bioavailability after dose adjustment is estimated to be considerably lower for TRANSMUCOSAL. In conjunction with other parameters, comparable *C*_max_ values may serve as an indication that INTRANASAL and TRANSMUCOSAL might result in comparable clinical efficacy for acute pain.

*C*_max_ point estimates for INTRANASAL are considerably higher when compared to dose-adjusted TRANSMUCOSAL. Intranasal administration delivers the drug first to the central blood compartment before it can cross the blood-brain barrier. This is supported by the nasal mucosa, which exhibits comparatively high blood circulation, even higher than the issue of the liver or muscles, and, as such, circumvents first-pass metabolism [39]. Also, the nasal administration route partly delivers the molecule directly to the site of action of the CNS [40]. Therefore, the intranasal administration of lower doses of opioids may potentially lead to similar therapeutic effects compared to other modes of administration [41]. In addition, the blunted peak exposure compared to intravenous fentanyl may also be favourable from a safety perspective.

INTRANASAL reached maximum concentrations (*t*_max_) in a shorter timeframe than TRANSMUCOSAL, confirming results from a previous study [17], although it still took more than three times longer to reach *t*_max_ compared with the intravenous investigational product. Excluding intravenous injection, intranasal administration of fentanyl is the fastest noninvasive administration route to reach *t*_max_ before lozenges, sublingual tablets and sprays, buccal tablets, and films, as well as transdermal delivery systems [18, 42]. The finding suggests and confirms that intranasal administration is principally beneficial for rapid acute pain relief.

Striebel et al. carried out various studies to assess the suitability of intranasal fentanyl for postoperative pain management [16, 24, 43, 44]. For these investigations, the team used the intravenous formulation of fentanyl (50 *μ*g/mL) administered intranasally in six puffs of 90 *μ*L each dose, totalling 27 *μ*g fentanyl in 0.54 mL (0.27 mL per nostril). In one randomised, double-blind study, the efficacy of intranasal versus intravenous demand-adapted titration (repeated dosing every five minutes until no longer required or requested) was tested in a standardised population of 42 patients with intense pain after surgery for lumbar intervertebral disk protrusion [43]. All patients were satisfied with the pain reduction achieved. The same treatment schedule was used in another study, but an unselected population of 112 patients with severe postoperative pain was assessed herein [44]. Adequate pain relief was achieved in 52 of 53 patients of the intranasal cohort and in all patients in the intravenous group.

The formulation investigated within this study contains 47 *μ*g of fentanyl per administration, and the bioavailability was determined in the magnitude of 77% (unadjusted), leading to around 36 *μ*g fentanyl being systemically available. Such an amount of systemically available fenantyl would exceed the amount of fentanyl patients received in the aforementioned Striebel studies. Therefore, the chosen concentration of 47 *μ*g fentanyl for intranasal administration appears sufficient for adequate postoperative pain relief.

## 4. Conclusion

Presented data strongly affirm the assumption that the developed and investigated intranasal formulation is principally suitable for intranasal PCA.

## Figures and Tables

**Table 1:** Characteristics of volunteers completing the study.

	*n* = 24 (12 males, 12 females)			
	Age (years)	Height (cm)	Weight (kg)	BMI (kg/m^2^)
Median	32.0	173.5	70.50	24.07
Mean	33.8	173.5	72.52	23.96
SD	10.22	9.97	11.683	1.893
Min	18	156	53.0	19.9
Max	51	194	101.8	27.0

**Table 2:** Pharmacokinetic parameters for single administration of INTRANASAL.

INTRANASAL (intranasal fentanyl, 140 *μ*L per puff, 47 *μ*g)—one puff										
	AUC_0-tlast_	AUC_0-∞_	AUC_expol%_	*C*_max_	C_last_	*t*_max_	*t*_1/2_	*t*_last_	*λ*_*z*_	MRT
N	24	23	23	24	24	24	23	24	23	23
Unit	h *∗* pg/ml	h *∗* pg/ml	%	pg/ml	pg/ml	h	h	h	1/h	h
Arith. Mean						0.21	12.18	10.71	0.13898	2.92
SD						0.078	9.513	2.647	0.20596	0.789
Min	296	323	3.69	111	10	0.10	0.71	4.00	0.02004	1.19
Median	571	748	23.33	370	15	0.20	8.05	12.00	0.08608	3.14
Max	846	1726	63.84	595	24	0.50	34.59	12.02	0.97349	4.02
Geom. Mean	515	766	25.09	338	16					
CV% <Geom. Mean	30.10	47.25	80.05	45.61	27.16					

AUC_0-tlast_: area under the plasma concentration versus time curve from dosing time to the last measurement time point with concentration value above the lower limit of quantitation; AUC_0-∞_: area under the plasma concentration versus time curve from dosing time to the infinite; AUC_expol%_: fraction of the total AUC due to extrapolated AUC; *C*_max_: maximum concentration in plasma; C_last_: concentration at the last time quantifiable point; *t*_max_: time to reach maximum drug concentration; *t*_1/2_: terminal half-life; *t*_last_: last quantifiable time point; *λ*_*z*_: apparent terminal elimination rate constant; MRT: mean residence time; SD: standard deviation; CV%: coefficient of variation.

**Table 3:** Pharmacokinetic parameters for single administration of INTRAVENOUS.

INTRAVENOUS (intravenous infusion, 10 mL, 50 *μ*g)—one short infusion (administration over 2 minutes)										
	AUC_0-tlast_	AUC_0-∞_	AUC_expol%_	*C*_max_	C_last_	*t*_max_	*t*_1/2_	*t*_last_	*λ*_*z*_	MRT
n	24	21	21	24	24	24	21	24	21	21
Unit	h *∗* pg/ml	h *∗* pg/ml	%	pg/ml	pg/ml	h	h	h	1/h	h
Arith. Mean						0.06	23.09	11.01	0.13205	27.13
SD						0.056	39.350	2.342	0.17853	51.293
Min	291	317	2.44	303	11	0.03	0.93	5.00	0.00430	1.14
Median	697	973	21.86	938	16	0.03	7.94	12.00	0.08726	7.46
Max	1230	7162	84.52	2035	39	0.25	161.03	12.05	0.74557	206.88
Geom. Mean	672	1111	22.72	886	18					
CV% geom. Mean	32.18	78.41	109.82	59.38	41.12					

AUC_0-tlast_: area under the plasma concentration versus time curve from dosing time to the last measurement time point with concentration value above the lower limit of quantitation; AUC_0-∞_: area under the plasma concentration versus time curve from dosing time to the infinite; AUC_expol%_: fraction of the total AUC due to extrapolated AUC; *C*_max_: maximum concentration in plasma; C_last_: concentration at the last time quantifiable point; *t*_max_: time to reach maximum drug concentration; *t*_1/2_: terminal half-life; *t*_last_: last quantifiable time point; *λ*_*z*_: apparent terminal elimination rate constant; MRT: mean residence time; SD: standard deviation; CV%: coefficient of variation.

**Table 4:** Significance level of comparison of means between routes of administration.

Comparison				
		*C*_max_	*t*_max_	AUC_0-tlast_
INTRANASAL	INTRAVENOUS	*p* < 0.0001	*p* < 0.0001	*p* < 0.0023
INTRANASAL	TRANSMUCOSAL	*p* < 0.1825	*p* < 0.0001	*p* < 0.0001
TRANSMUCOSAL	INTRAVENOUS	*p* < 0.0001	*p* < 0.0001	*p* < 0.0001

**Table 5:** Pharmacokinetic parameters for single administration of TRANSMUCOSAL.

TRANSMUCOSAL (oral transmucosal fentanyl citrate, 1 lozenge, 200 *μ*g)—one lozenge (administration over 15 minutes)										
	AUC_0-tlast_	AUC_0-∞_	AUC_expol%_	*C*_max_	C_last_	*t*_max_	*t*_1/2_	*t*_last_	*λ*_*z*_	MRT
N	24	21	21	24	24	24	21	24	21	21
Unit	h *∗* pg/ml	h *∗* pg/ml	%	pg/ml	pg/ml	h	h	h	1/h	h
Arith. Mean						1.20	12.09	11.76	0.10910	3.74
SD						0.763	16.190	1.226	0.09975	0.567
Min	434	457	4.91	164	11	0.42	1.35	6.00	0.00899	1.81
Median	1102	1452	26.50	326	37	1.04	7.86	12.00	0.08824	3.83
Max	1925	7017	80.98	535	77	2.52	77.09	12.05	0.51173	4.53
Geom. Mean	1106	1605	23.89	310	34					
CV% geom. Mean	32.86	61.49	64.02	29.58	47.21					

AUC_0-tlast_: area under the plasma concentration versus time curve from dosing time to the last measurement time point with concentration value above the lower limit of quantitation; AUC_0-∞_: area under the plasma concentration versus time curve from dosing time to the infinite; AUC_expol%_: fraction of the total AUC due to extrapolated AUC; *C*_max_: maximum concentration in plasma; C_last_: concentration at the last time quantifiable point; *t*_max_: time to reach maximum drug concentration; *t*_1/2_: terminal half-life; *t*_last_: last quantifiable time point; *λ*_*z*_: apparent terminal elimination rate constant; MRT: mean residence time; SD: standard deviation; CV%: coefficient of variation.

**Table 6:** Parametric point estimates for AUC_0-tlast_ after dose adjustment.

AUC_0-tlast_						
Comparison		PE (%)	90% CI (%)		CV_ANOVA_ (%)	Power (%)
INTRANASAL^*∗*^	INTRAVENOUS	74.70	69.01	80.87	16.10	99.74
INTRANASAL	TRANSMUCOSAL	45.27	41.64	49.23	17.00	99.54
TRANSMUCOSAL	INTRAVENOUS	165.01	152.83	178.15	15.60	99.83
TRANSMUCOSAL^*∗*^	INTRAVENOUS	41.25	38.21	44.54	15.60	99.83
INTRANASAL	TRANSMUCOSAL^*∗∗*^	198.18	182.26	215.49	17.40	99.54

^*∗*^Dose adjusted to 50 *μ*g fentanyl. ^*∗∗*^dose adjusted to 47 *μ*g fentanyl.

**Table 7:** Parametric point estimates for *C*_max_ after dose adjustment.

*C*_max_						
Comparison		PE (%)	90% CI (%)		CV_ANOVA_ (%)	Power (%)
INTRANASAL^*∗*^	INTRAVENOUS	37.22	30.15	45.94	44.50	54.13
INTRANASAL^*∗*^	TRANSMUCOSAL	105.97	94.47	118.88	23.50	94.00
TRANSMUCOSAL	INTRAVENOUS	35.12	28.06	43.96	47.70	49.72
TRANSMUCOSAL^*∗*^	INTRAVENOUS	8.78	7.02	10.99	45.60	49.72
INTRANASAL	TRANSMUCOSAL^*∗∗*^	463.88	424.64	506.73	17.90	94.00

^*∗*^Dose adjusted to 50 *μ*g fentanyl. ^*∗∗*^dose adjusted to 47 *μ*g fentanyl. AUC_0-tlast_: area under the plasma concentration versus time curve from dosing time to the last measurement time point with concentration value above the lower limit of quantitation; *C*_max_: maximum concentration in plasma; PE: parametric point estimate; CI: confidence interval; CV%: coefficient of variation.

**Table 8:** Summary of adverse events.

	Total		Naltrexone		INTRANASAL		INTRAVENOUS		TRANSMUCOSAL	
	Absolute	Relative (%)	Absolute	Relative (%)	Absolute	Relative (%)	Absolute	Relative (%)	Absolute	Relative (%)
Adverse events (AEs)	38	100								
Volunteers without AEs	10	37								
Volunteers with AEs	17	63								

*Maximum intensity*										
Mild	31	82	3	60	11	79	12	100	5	71
Moderate	7	18	2	40	3	21	0	0	2	29
Severe	0	0	0	0	0	0	0	0	0	0

*Drug relationship*										
Probable	0	0	0	0	0	0	0	0	0	0
Possible	14	37	0	0	7	50	3	25	4	57
No causal relationship	11	29	3	60	2	14	5	42	1	14
Unclassified	13	34	2	40	5	36	4	33	2	29

*Outcome*										
Recovered	38	100	5	100	14	100	12	100	7	100
Recovered with sequelae	0	0	0	0	0	0	0	0	0	0
Not yet recovered	0	0	0	0	0	0	0	0	0	0
Death	0	0	0	0	0	0	0	0	0	0
Unknown	0	0	0	0	0	0	0	0	0	0

*Seriousness*										
Serious	0	0	0	0	0	0	0	0	0	0
Not serious	38	100	5	100	14	100	12	100	7	100

*Frequency of top 5 AEs*										
Headache	5	13	0	0	2	14	1	8	2	29
Nausea	7	18	0	0	1	7	4	33	2	29
Vomiting	3	8	1	20	2	14	0	0	0	0
Tiredness	2	5	0	0	2	14	0	0	0	0
Abdominal pain	2	5	0	0	1	7	1	8	0	0

## Data Availability

The data used to support the findings of this study are available from the corresponding author upon request and approval by the rights owner.
